# Diagnosing lung involvement in inflammatory rheumatic diseases—Where do we currently stand?

**DOI:** 10.3389/fmed.2022.1101448

**Published:** 2023-01-11

**Authors:** Tobias Hoffmann, Peter Oelzner, Ulf Teichgräber, Marcus Franz, Nikolaus Gaßler, Claus Kroegel, Gunter Wolf, Alexander Pfeil

**Affiliations:** ^1^Department of Internal Medicine III, Jena University Hospital–Friedrich Schiller University Jena, Jena, Germany; ^2^Institute of Diagnostic and Interventional Radiology, Jena University Hospital–Friedrich Schiller University Jena, Jena, Germany; ^3^Department of Internal Medicine I, Jena University Hospital–Friedrich Schiller University Jena, Jena, Germany; ^4^Department of Pathology, Jena University Hospital–Friedrich Schiller University Jena, Jena, Germany

**Keywords:** inflammatory rheumatic disease, interstitial lung disease, pulmonary function tests, high-resolution computed tomography (HRCT), bronchoalveolar lavage (BAL), PET/CT

## Abstract

Lung involvement is the most common and serious organ manifestation in patients with inflammatory rheumatic disease (IRD). The type of pulmonary involvement can differ, but the most frequent is interstitial lung disease (ILD). The clinical manifestations of IRD-ILD and severity can vary from subclinical abnormality to dyspnea, respiratory failure, and death. Consequently, early detection is of significant importance. *Pulmonary function test* (PFT) including diffusing capacity of the lungs for carbon monoxide (DLCO), and *forced vital capacity* (FVC) as well as high-resolution computed tomography (HRCT) are the standard tools for screening and monitoring of ILD in IRD-patients. Especially, the diagnostic accuracy of HRCT is considered to be high. Magnetic resonance imaging (MRI) and positron emission tomography/computed tomography (PET/CT) allow both morphological and functional assessment of the lungs. In addition, biomarkers (e.g., *KL-6, CCL2*, or MUC5B) are being currently evaluated for the detection and prognostic assessment of ILD. Despite the accuracy of HRCT, invasive diagnostic methods such as *bronchoalveolar lavage* (BAL) and lung biopsy are still important in clinical practice. However, their therapeutic and prognostic relevance remains unclear. The aim of this review is to give an overview of the individual methods and to present their respective advantages and disadvantages in detecting and monitoring ILD in IRD-patients in the clinical routine.

## Key messages

• Patients with inflammatory rheumatic diseases (IRD) are at particular risk of developing pulmonary diseases such as interstitial lung disease (ILD) which is associated with a increased morbidity and mortality
• Pulmonary function tests (PFT) with measurements of FVC and DLCO as well as high-resolution computed tomography (HRCT) are the current standards for screening and monitoring ILD in IRD
• Cardiopulmonary stress tests are, additional to PFT, suitable for the assessment of prognosis and evaluating the response to therapy in ILD
• New imaging technologies such as magnetic resonance imaging (MRI) and positron emission tomography (PET) with computed tomography (PET/CT) can evaluate both morphological and functional features
• The value of invasive methods like bronchoalveolar lavage (BAL) and lung biopsy has not yet been adequately investigated
• Numerous biomarkers with good sensitivity and specificity exist for detection and prognostic evaluation but have not yet found their way into clinical routine

## Introduction

Inflammatory rheumatic diseases (IRD) belong to the wide spectrum of immune-mediated inflammatory diseases (IMID) comprising inflammatory joint diseases, connective tissue diseases (CTD), myositis as well as vasculitis (1–6).

Many IRD present with complex clinical pictures, involving other tissues: In total, 53% of IRD-patients show an organ involvement at initial diagnosis (7) with a predominant affection of the lungs, followed by the heart and kidneys (8–13). Patients with CTD, myositis/dermatomyositis, and vasculitis are particularly susceptible for solid organ manifestations.

Pulmonary manifestations present special diagnostic and therapeutic challenges and are associated with a significant morbidity and mortality in IRD-patients. The most common clinical pattern of lung illness encountered in IRD is interstitial lung disease (ILD) (8, 9, 11), ranging between 12.5 and 30.8% at the onset of CTD, 66.7–83.3% with vasculitis and 16.7–100.0% with myositis (7). In addition, lung diseases are also found in association with rheumatoid arthritis (RA) with a life-time risk of developing ILD of 7.7% (14, 15).

The clinical manifestations and severity of IRD-ILD can vary from subclinical abnormality to dyspnea, respiratory failure, and death (16–18).

International guidelines for the management and diagnostic of IRD-ILD do not exist. There is only for patients with systemic sclerosis (SSc) an European evidence-based consensus statement available (19). According to the current literature and international guidelines, parallels can only be drawn with idiopathic pulmonary fibrosis (IPF), for which high-resolution computed tomography (HRCT) is the diagnostic gold standard (20–22). Furthermore, in different studies pulmonary function tests (PFT), bronchoalveolar lavage (BAL), and biomarkers [e.g., Krebs von den Lungen 6 (KL-6), chemokine (C-C motif) ligand 2 (CCL2), or mucin 5B (MUC5B)] were also discussed as potential diagnostic tools (23–25).

Patients at early stages of IRD are often asymptomatic with an HRCT-finding of ground-glass opacity (GGO) and reticulation (26, 27). In established ILD and in the presence of pulmonary symptoms, HRCT often reveals a specific pattern like usual interstitial pneumonia (UIP) or non-specific interstitial pneumonia (NSIP) (28, 29). Finally, the autoimmune-mediated lung injury can lead to chronic progressive fibrosing ILD as final manifestation (30, 31).

The frequency and significant increased morbidity and mortality of IRD-ILD as well as the availability of new therapeutic options [e.g., nintedanib and tocilizumab (approved for SSc-ILD by the FDA)] underline the importance of an early diagnosis. However, the optimal use of the different diagnostic tools in the clinical routine is not yet clear defined (32, 33).

Therefore, the present review aims to provide an overview of the various diagnostic tools and their value in detecting ILD and offers an evaluation of these procedures for the long-term follow up. For this an overview is given in Table 1 with advantages and disadvantages of different methods in diagnosing ILD in IRD-patients. Moreover, we supplement a case-report with pulmonary involvement in systemic lupus erythematosus to this review to illustrate and demonstrate the possible and complex pulmonary diagnostic in IRD (see Supplementary material).

**TABLE 1:** Advantages and disadvantages of different methods in diagnosing ILD in IRD-patients.

	Examination		Advantages (+) and disadvantages (−)	
Non-invasive	Pulmonary function test	FVC	+	Good working for monitoring in CTD and myositis
			−	Suitable to only a limited extend for vasculitis
		DLCO	+	Good working for screening in CTD and myositis
			(+)	Maybe usable for vasculitis
		TLC	+	Good working for screening in myositis
			−	Not ideal for screening or monitoring ILD in CTD or vasculitis
	Cardiopulmonary stress tests		+	Statement about the patient’s response to physical stress
			+	Good working for screening and monitoring ILD
			(−)	Moderately evaluated for ILD in IRD
			(−)	Reliability only with adherence to strict recommendations
			−	Higher time effort
Imaging	Chest X-ray		+	High sensitivity and specificity in moderate to severe ILD
			−	Low sensitivity in detecting early ILD
			−	No value in monitoring ILD
			−	No classification in CT pattern
			−	Radiation exposure
	HRCT		+	Gold standard
			+	Highly sensitive in detecting morphological abnormalities classification of CT pattern
			+	Easy access
			+	Specificity remains unclear, especially in case of unclassifiable changes
			−	Radiation exposure
	MRI		+	No radiation exposure
			+	Hints for differentiation between inflammation and fibrosis of predominant lesions
			+	Monitoring treatment possible
			−	Lower spatial resolution than HRCT
			−	High level of experience necessary
	PET/CT		+	Investigation of the cellular metabolism and morphological changes at the same time
			−	High level of experience necessary
			−	Radiation exposure
Blood	Bio-markers	Auto-antibodies	+	Known association to risk and progression of ILD
			+	Already used in clinical practice
		Blood biomarkers	+	Good correlation between ILD and serum levels
			+	Also useful for prognostic evaluation
			+	Could be well integrated into an algorithms in the future
			−	So far only experimental
		Non-blood biomarkers	−	Only limited data available
Invasive	BAL		+	Cansupport clinical diagnosis and differential diagnosis
			+	Invasive method with the lowest complication rate
			(+)	Should be interpreted in clinical context
			−	No known prognostic or therapeutic value
	Biopsy	Open surgical procedure	+	Correlation with HRCT and pathology
		(e.g., wedge resection)	+	Can support clinical diagnosis and differential diagnosis
			+	More representative than transbronchial biopsy
			−	High complication rate
		Trans-bronchial	+	Correlation with HRCT and pathology
			+	Can support clinical diagnosis and differential diagnosis
			(+)	Moderate complication rate
			−	Less representative than open surgery
Combinations	PFT and Imaging		+	For screening: By combining chest X-ray and PFT, specificity could be increased
			−	For monitoring: No established algorithms available
			+	Combination could be the key in screening and monitoring ILD in IRD
	PFT, imaging, invasive diagnostic methods, and biomarkers		+	Combination could be the key in screening and monitoring ILD in IRD
			−	No established algorithms available

## Clinical signs and symptoms

There are some clinical signs and symptoms that are more likely to be associated with ILD. They may support the suspected diagnosis of ILD and can also justify further investigation. A typical clinical sign for fibrosis of the lung in clinical examination are bibasilar inspiratory crackles (sclerosiphonia), but patients also frequently report dyspnea and cough (21, 34). Distler et al. (35) was able to show that patients with SSc ILD had a mean functional assessment of cancer therapy (FACIT) dyspnea score of 47.01 ± 9.64. Lin et al. (36) describe cough and dyspnea as a risk factor for ILD in Sjögren’s syndrome. Moreover, Hoffmann et al. (27) demonstrated that also at initial diagnosis of IRD-ILD, there is significantly more dyspnea and sclerosiphonia compared with patients without ILD. However, because there are other causes for these signs, they are not necessarily specific for ILD (37).

Digital clubbing is a typical clinical sign of pulmonary fibrosis (PF), but the specificity is low, as many other diseases can also show such abnormalities (38). For ILD in IRD, only a few studies/case reports exist, but no systematic analysis for the presence of digital clubbing. Van Manen et al. (39) describe in a cohort of ILD in IRD 37% of patients with digital clubbing, assessed by a physician. In addition, we know certain pathognomonic findings in IRD increasing the risk of ILD, such as mechanic’s hands (myositis) or Gottron’s papules (dermatomyositis) (40–42). But the risk of ILD, depending on the clinical sign, is very heterogeneous between the IRD. In SSc male sex and ethnicity are considered as independent risk factors for progressive SSc-ILD (19). Moreover, Lin et al. (36) reported Raynaud’s phenomenon as a risk factor for ILD in newly diagnosed Sjögren’s syndrome. Knowing these symptoms and clinical signs, any clinician can take advantage of this knowledge and use it without additional equipment. Furthermore, it should be emphasized that pulmonary asymptomatic patients may already have ILD on HR-CT in the setting of initial diagnosis of IRD (27).

## Pulmonary function test

The presence and progression of ILD are routinely monitored using PFT. Various parameters can be measured through different examinations like spirometry, body plethysmography or diffusing capacity of the lungs for carbon monoxide (DLCO) (43–45). Spirometry is the most common PFT (43–45). The most relevant measurements are the forced vital capacity (FVC), which describes the total air volume that can be exhaled after a full inspiration, and the forced expiratory volume in 1 s (FEV_1_), which is the expiratory volume in the first second of an FVC maneuver (44). Supplementary information is provided by body plethysmography which allows to determine total lung capacity (TLC), lung residual volume (RV), and airway resistance (46, 47). Additionally, DLCO is used to estimate the lungs’ ability to transfer gas from the inspired air to the bloodstream (48).

In rheumatology, many studies evaluated the relevance of PFT as surrogate markers for ILD in IRD; we summarized the most relevant literature in Table 2. Various parameters can be considered, with the focus on FVC and DLCO in the current literature. Although data are limited, there is evidence, that we have to differentiate between monitoring a known ILD and screening for newly onset ILD in IRD (27). In addition, it is necessary to distinguish between the different IRD-related diseases (27).

**TABLE 2:** Values, sensitivities, specificities, positive (PPV) and negative predictive values (NPV) for different parameters in pulmonary function test (PFT) for screening and monitoring interstitial lung disease (ILD) in patients with inflammatory rheumatic diseases (IRD) (cut-off < 80%).

Parameters		Values	SD	Sensitivity	Specificity	PPV/Positive LR	NPV/Negative LR
CTD	FVC	69.9–98% (27, 49, 51, 60, 107–109)	±18.5–21.4% (27)	37.5–69% (27, 49–51, 109)	68–78.7% (27, 50, 51)	70–86%/2.23 (27)	49–61%/0.67 (27)
	TLC	65–101% (27, 49, 51, 108)	±19.8–20.3% (27)	32.1–46% (27, 51)	77–94.6% (27, 51)	74%/5.94 (27)	51%/0.72 (27)
	FEV_1_	77.6–82.0% (27, 51, 108, 109)	±18.6–23.4% (27)	49.2% (27)	82.0% (27)	2.73 (27)	0.62 (27)
	DLCO	47.5–78% (27, 49, 51, 60, 107–109)	±16.8–22.2% (27)	80–92% (27, 50, 51)	32–51% (27, 50, 51)	66–77%/1.53 (27)	63–68%/0.36 (27)
	TLCO	75.0–86.2% (27, 108)	±19.1–19.6% (27)	57.6% (27)	67.8% (27)	1.79 (27)	0.63 (27)
Vasculitis	VC	67% (52)					
	FVC			41% (53)			
	TLC	102% (52)		36% (53)			
	FEV_1_	91% (52)		55% (53)			
	DLCO	88% (52)		36.0–66.7% (27, 53)	45.8% (27)		
	TLCO	88% (52)					
Myositis	VC	66.2–82.2% (54, 57)	±8.2–9.2%	75% (57)			
	FVC	60.5–70% (55, 56)	±13.7%				
	TLC	77.0–77.4% (55, 57)		75% (57)			
	FEV_1_	67.2–88.3% (54, 56)	±2.7–13.8%	91.7% (27)	45.8% (27)		
	DLCO	50.5–59.9% (54–56)	±3.1–15.7%	88–94% (55, 56)			
	TLCO	62.4% (57)					

### Pulmonary function test for detecting/Screening for newly onset interstitial lung disease in inflammatory rheumatic disease

Various studies suggest that an impaired DLCO (<80%) may have a predictive value for the development of ILD. Thus, Suliman et al. (49) showed that the DLCO (<80%) was the only frequently pathological parameter in the PFT, compared to FVC or TLC. This in accordance with Showalter et al. (50) demonstrating a sensitivity and specificity of 92.0 and 32.0% for DLCO < 80%, with the highest negative predictive value (NPV) of 63% in patients with SSc. Bernstein et al. (51) reported a sensitivity of 80.0% and specificity of 51.0% with NPV of 68% in detecting ILD by DLCO < 80% in early diffuse SSc. However, TLC shows only sensitivities and specificities intermediate between those of FVC and DLCO (49, 51). Therefore, it can be assumed that TLC is neither suitable for screening nor monitoring of ILD.

In patients with anti-neutrophil cytoplasmic antibody (ANCA)-associated vasculitis (AAV), Newall et al. (52) demonstrated no significant differences in FVC, TLC or FEV_1_ between patients with or without ILD. Furthermore, the study yielded a reduced DLCO in ANCA vasculitis patients with ILD (52). Rosenberg et al. (53) showed sensitivities of 55% (FEV_1_) and 41% (FVC). Therefore, PFT based on FVC, TLC, and FEV_1_ alone do not seem not to be valid surrogate parameters for ILD in AAV-patients.

This also applies for patients with myositis, although the study populations are rather small, which makes it difficult to obtain a conclusion. Ideura et al. (54) reported reduced DLCO- and FVC-values and a normal FEV_1_ in patients with amyopathic dermatomyositis. The data of a 10-year retrospective analysis published by Chua et al. (55) showed a restrictive ventilatory defect in the majority (79%) of patients with idiopathic inflammatory myositis (IIM) with mean baseline values of FVC and TLC of 70 and 77%, respectively. 94% of patients had a baseline DLCO of <80%. This is in accordance with the results of Won Huh et al. (56), revealing a restrictive defect (92.9%) as the most common PFT abnormality, followed by a low DLCO (88.0%) in patients with polymyositis/dermatomyositis (PM/DM); 80% had a restrictive defect with a reduced DLCO. Fathi et al. (57) observed also restrictive changes on PFT and reduced DLCO in almost all PM/DM-patients with radiological evidence of ILD. Overall, in patients with myositis, restrictive patterns (especially TLC) and a reduced DLCO seem to be important for diagnosing ILD.

### Pulmonary function test for monitoring interstitial lung disease in inflammatory rheumatic disease

As shown in a systematic review by Caron et al. (24), FVC (% predicted) is the most commonly used surrogate marker in studies evaluating ILD progression in SSc-patients. FVC is widely used as primary endpoint in studies, but is also a generally recognized sign of disease progression (decline of 10%) (21, 58, 59). One of the main reasons for FVC’s current popularity in SSc is the fact that FVC is believed to be more specific than DLCO for ILD which can be more useful for monitoring ILD (24). Suliman et al. (49) and Showalter et al. (50) demonstrated a sensitivity and specificity of 37.5–69.0% and 73.0–92.0%, respectively, for FVC < 80%. According to the evidence-based European consensus which has been developed by a panel of 27 European pulmonologists, rheumatologists, and internists with expertise in SSc-ILD, FVC, and DLCO are considered to be useful parameters for evaluation disease progression in SSc (19). In addition, multivariate analyses of a prospective cohort study with SSc-patients identified baseline DLCO as one of the predictors of no fibrosis at follow-up and FVC as predictors of >20% fibrosis at follow-up (60). However, there is still a lack of large ILD studies in IRD, especially for myositis and vasculitis.

## Cardiopulmonary stress tests

Pulmonary function test is a static examination which does not allow any statement to be made about the patient’s response to physical stress. For many decades, cardiopulmonary stress tests such as the 6-min walk test (6MWT) and cardiopulmonary exercise test (CPET) have been used to evaluate the performance of the patients.

According to American Thoracic Society (ATS) guideline the 6MWT and CPET evaluates the global and integrated responses of all the systems involved during exercise (pulmonary, cardiovasculary, systemic and peripheral circulation, neuromuscular system, muscle metabolism) (61, 62). The 6MWT assesses the submaximal level of functional capability, whereas the CPET evaluates submaximal and peak exercise response (61, 62).

The ATS and European Respiratory Society (ERS) positions paper emphasize the benefits of 6MWT in assessing prognosis, evaluating response and functional exercise capacity in respiratory disease (63). Especially in chronic obstructive pulmonary disease (COPD), a reduced 6MWT was associated with an increased risk of hospitalization and mortality (63). Similar, results were demonstrated for ILD by the ATS/ERS systematic literature review (63). Even CPET is useful in verifying early ILD regarding the detection of minor pulmonary gas exchange abnormalities and therapy monitoring in established ILD (62, 64, 65). Additionally, Keogh et al. (65) could show that CPET can reveal alveolar dysfunction in the presence of normal resting parameter. In summary, 6MWT and CPET represent additional diagnostic (besides PFT and imaging), providing information to improve the care and therapy of patients with ILD.

## Imaging

Unlike PFT, imaging techniques allow an overview of the morphologic features of the lungs. Modern techniques such as magnetic resonance imaging (MRI) and positron emission tomography/computed tomography (PET/CT) have broadened the spectrum of “classical methods” such as X-ray or HRCT (66).

### Chest X-ray

**FIGURE 1:**
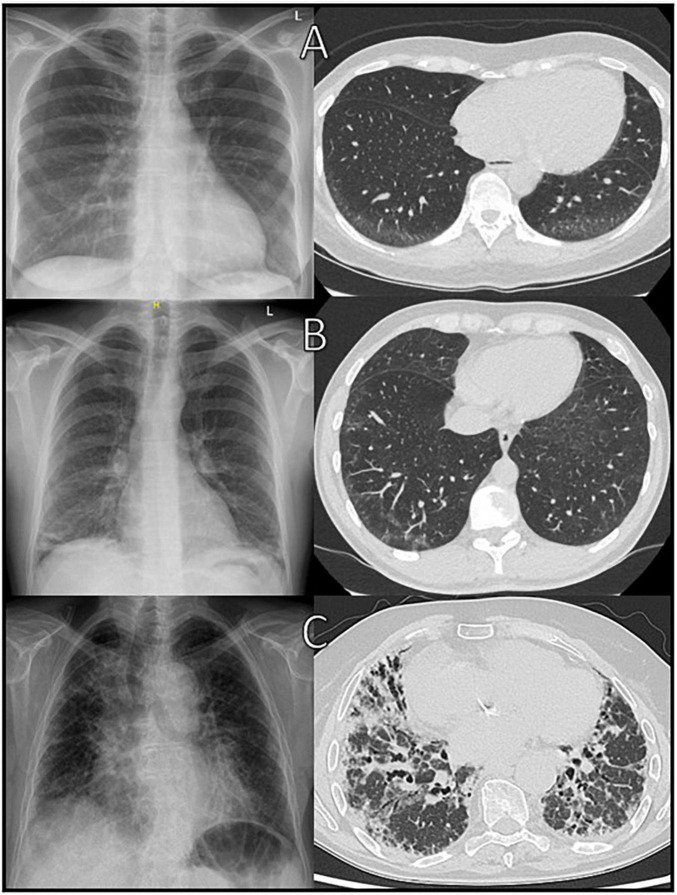
Comparison of chest X-ray with HRCT at initial diagnosis of ILD. **(A)** Early Sharp syndrome with ground-glass opacities in HRCT and inconspicuous chest X-ray, **(B)** early systemic sclerosis with ground-glass opacities, reticulations (early NSIP-pattern), and small consolidations in chest X-ray and **(C)** end-stage Sjögren’s syndrome with distinct consolidations in chest X-ray and beginning UIP-pattern in HRCT.

Chest X-ray is the most simple and cost-effective method for morphological assessment of the lungs. In everyday clinical practice, it continues to be of value for overview imaging of the lungs or to exclude/confirm infections. However, chest radiographs are insensitive to early changes and may appear normal despite respiratory function test abnormalities and are therefore no longer of any value in diagnosing ILD (67) (see Figure 1).

### High-resolution computed tomography

High-resolution computed tomography is the gold standard for the detection, characterization, and monitoring of ILD (19, 67). According to the Fleischner Society, the term interstitial lung abnormalities (ILA) refers to specific CT findings that are potentially compatible with ILD in patients without clinical suspicion of the disease (66). ILA have been described as non-dependent abnormalities affecting more than 5% of any lung zone (upper, middle, and lower lung zones are demarcated by the levels of the inferior aortic arch and right inferior pulmonary vein) (66). ILA include the following radiological signs (66):

• Ground-glass opacity
• Reticular abnormalities
• Lung distortion
• Traction bronchiectasis
• Honeycombing
• Non-emphysematous cysts

These parenchymal findings can be classified according to the definition by the Fleischner Society and the ATS/ERS (see Tables 3, 4) (67–69) in consideration of the differential diagnosis of idiopathic interstitial pneumonia [e.g., hypersensitivity pneumonia (HP)] according to ATS/ERS (details see Table 5) (67–69). If ILA present less than 5% of any lung zone, they are defined as indeterminate ILA. Depending on the cohort, the prevalence of indeterminate ILD ranges from 32 to 59% (70). According to Putman et al. (70), ILA and indeterminate ILA are associated with a greater all-cause mortality. The transition between ILA and ILD is not clearly defined and should be determined by clinical parameters. Especially in IRD, even small ILA is considered as ILD (66). The diagnostic follow up of patients with indeterminate ILA remains completely unclear, because especially in early cases, no extensive pulmonary changes can be expected, but patients could benefit to a greater extent from therapy.

**TABLE 3:** Radiographic and histologic features of major idiopathic interstitial pneumonias (IIP) according to the classification of the American Thoracic Society (ATS) and European Respiratory Society (ERS) (68, 69).

Idiopathic interstitial pneumonia (IIP)	Grading	Radiographic features/Distribution	Histologic features
Usual interstitial pneumonia (UIP)	Chronic fibrosing	• Basal-predominant reticular abnormality with volume loss • Honeycombing • Peripheral, subpleural, basal	• Dense fibrosis with frequent honeycombing • Fibroblastic foci • Patchy lung involvement • Subpleural und paraseptal distribution
Non-specific interstitial pneumonia (NSIP)	Chronic fibrosing	• Ground-glass and reticular opacity • Peripheral, subpleural, basal, symmetric	• Cellular pattern: mild to moderate interstitial chronic inflammation • Fibrosing pattern: dense or loose interstitial fibrosis lacking the temporal heterogeneity pattern and/or patchy features of UIP, chronic inflammation can appear
Cryptogenic organizing pneumonia (COP)	Acute/subacute IIP	• Patchy bilateral consolidation • Subpleural, peribronchial	• Intraluminal organizing fibrosis in distal airspaces • Patchy distribution • Preservation of lung architecture • Uniform temporal appearance • Mild interstitial chronic inflammation
Acute interstitial pneumonia (AIP)	Acute/subacute IIP	• Progressive diffuse ground-glass density/consolidation • Diffuse	• Alveolar septal thickening due to organizing fibrosis • Airspace organization • Hyaline membranes • Diffuse distribution
Respiratory bronchiolitis-associated interstitial lung disease (RB-ILD)	Chronic fibrosing	• Bronchial wall thickening, ground-glass opacity • Diffuse	• Bronchocentric alveolar macrophage accumulations • Mild bronchiolar fibrosis and chronic inflammation
Desquamative interstitial pneumonia (DIP)	Chronic fibrosing	• Ground-glass opacity • Lower zone, peripheral predominance	• Prominent accumulation of alveolar macrophages • Mild to moderate fibrotic thickening of alveolar septa • Mild interstitial chronic inflammation • Uniform involvement

**TABLE 4:** Radiographic and histologic features of rare, unclassifiable, and other idiopathic interstitial pneumonias (IIP) according to the classification of the American Thoracic Society (ATS) and European Respiratory Society (ERS) (68, 69, 110).

Idiopathic interstitial pneumonia (IIP)		Radiographic features/Distribution	Histologic features
Rare IIP	Lymphoid interstitial pneumonia (LIP)	• Reticular opacities, nodules • Cyst formation • diffuse	• Infiltrates compromise mostly T lymphocytes • Frequent lymphoid hyperplasia • Predominantly alveolar septal distribution • Diffuse infiltration
	Pleuroparenchymal fibroelastosis (PPFE)	• Dense subpleural areas of airspaces consolidation with traction • upper lobe volume loss • Architectural distortion • Subpleural cyst	• Mild changes of PPFE or other patterns such as UIP
Rare histologic patterns	Acute fibrinous and organizing pneumonia (AFOP)	• Bilateral basal opacities and areas of consolidation	• Intraalveolar fibrin deposition and associated organized pneumonia • Hyaline membranes are absent
	Bronchiolocentric patterns of interstitial pneumonia	• Not well characterized	• Bronchiolocentric fibroinflammatory changes
	Unclassifiable idiopathic interstitial pneumonia	• Final diagnosis can not be achieved after multidisciplinary discussion • Often overlap of histologic patterns • Management should be based on the most probable diagnosis	
	Interstitial pneumonia with autoimmune features (IPAF)	• No specific radiographic or histologic feature • Classification criteria: 1. Presence of an interstitial pneumonia, by HRCT or surgical lung biopsy and 2. Exclusion of alternative etiologies and 3. Does not meet criteria of a defined CTD and 4. At least one feature from at least two of three domains (clinical, serological, morphological)	

**TABLE 5:** Radiographic and histologic features of important differential diagnosis according to the classification of the American Thoracic Society (ATS) and European Respiratory Society (ERS) (68, 69).

Differential diagnosis	Radiographic features/Distribution	Histologic features
Hypersensitivity pneumonia (HP)	• Centrilobular nodules • Mosaic air-trapping • Upper lobe distribution	• Bronchiolocentric distribution • Poorly formed granulomas • Consideration of specific circulating IgG antibodies (up to 30% of patients have no identifiable exposure)
Familial interstitial pneumonia (FIP)	• Cases remain classified as idiopathic interstitial pneumonia (IIP) • Indistinguishable in radiographic and histologic features to non-familial IIP	

**FIGURE 2:**
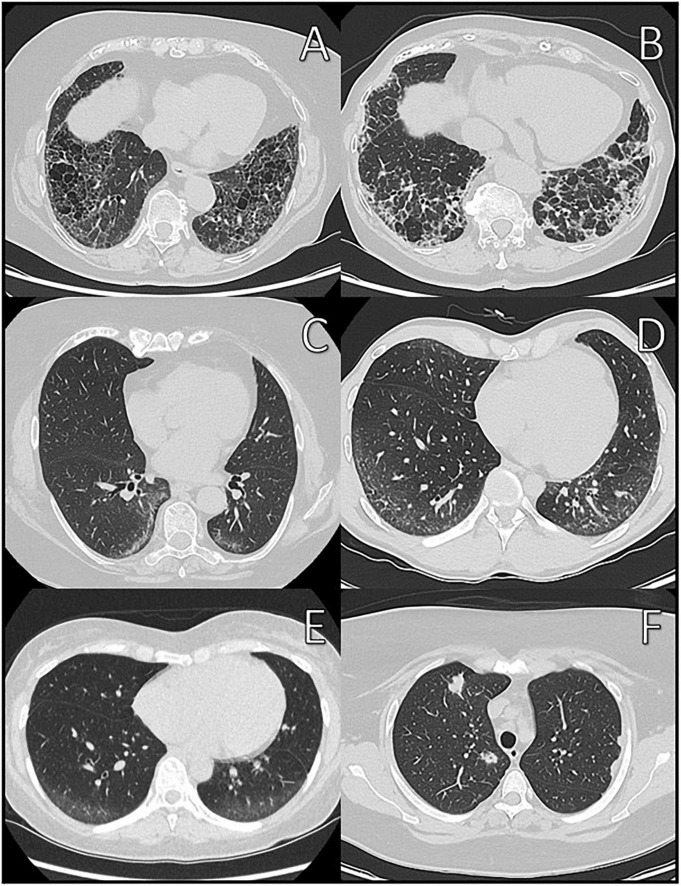
Different CT-patterns in patient with IRD at initial diagnosis. **(A)** Usual interstitial pneumonia (UIP) in polymyositis; **(B)** probable UIP in systemic sclerosis; **(C,D)** non-specific interstitial pneumonia (NSIP) in Sharp syndrome; **(E)** pure ground-glass opacities in Sharp syndrome; and **(F)** granuloma/proliferations in granulomatosis with polyangiitis.

The most common HRCT-patterns in IRD are NSIP and UIP, depending on the underlying immunologically mediated systemic disorder (28) (see Figures 1, 2A–D, F). According to Goldin et al. (26), among other changes pure ground-glass opacities (pGGO) and PF are the most common HRCT scan findings in patients with symptomatic SSc (see Figure 2E). The extent of PF seen on HRCT scans was significantly negatively correlated with FVC (*r* = −0.22), DLCO (*r* = −0.44), and TLC (*r* = −0.36). A positive correlation was revealed between pGGO and the increased number of acute inflammatory cells found in BAL fluid (*r* = 0.28). In addition, Remy-Jardin observed that areas of ground-glass attenuation are a reliable indicator of inflammation, as shown in histologic evaluations at open lung biopsy (71). Because differentiation from other causes of GGO such as pulmonary edema, alveolar hemorrhage, NSIP, and hypersensitivity pneumonitis can be challenging, consideration of relevant clinical information such as the chronicity of symptoms, the patient’s immune status, and pre-existing medical conditions is essential (72).

It is difficult to define sensitivity and specificity of HRCT in detecting ILD in IRD-patients. Due to the high resolution, HRCT as a single modality has a very high sensitivity of almost 100% for detecting ILD (27). However, the enhanced image resolution can also result in lower specificity due to unspecific findings. For instance, Hoffmann et al. (27) presented a specificity of 55.3% in detecting ILD in newly diagnosed IRD due to unspecific findings or other diseases. According to the Fleischner Society and Putman et al. (70), the specificity of HRCT may not be 100% and not all changes (ILA and indeterminate ILA) should be considered as being related to ILD. Further invasive diagnostic procedures would be necessary for differentiation.

In conclusion, HRCT is an important diagnostic tool in rheumatology. The evidence-based European consensus statements for identification and management of ILD in SSc recommend that SSc-patients should be screened for ILD using HRCT, particularly if they are showing one or more risk factors (19). In addition, the majority of studies regard HRCT generally as the gold standard for the diagnosis of ILD in IRD (49, 50, 55, 57, 73–75).

### Magnetic resonance imaging

Currently, MRI of the lungs is not used in clinical routine for diagnosing ILD, but there were some initial studies showing the potential of MRI in ILD. Although the spatial resolution on MRI is lower than on HRCT, it provides satisfactory results with a sensitivity and specificity of 89 and 91%, respectively, in the detection of ILD in direct comparison, but it can differ based on the technique used (76–78).

In addition, there are studies showing that MRI can differentiate between inflammation- and fibrosis-predominant lesions. Yi et al. (79) correlated biopsies with MRI and demonstrated an early enhancement pattern (82%) on dynamic studies in inflammation-predominant biopsy sites.

Overall, a better assessment and monitoring of ILD in IRD, maybe in combination with HRCT, could be achieved with MRI in the future. Furthermore, there is no risk of exposure to radiation during MRI, which seems to be an advantage for the use of monitoring ILD-IRD and treatment effects.

### Positron emission tomography/Computed tomography

The addition of PET to CT offers the ability to non-invasively investigate cellular metabolism *in vivo* and imaging of fine structural details of lung parenchyma (80).

The results of the first study using of ^18^F-FDG PET/CT for the investigation of IPF and other diffuse parenchymal lung disease (DPLD) indicate that increased glucose metabolism is associated with both the ground-glass and the reticulation/honeycombing HRCT lung changes (67). In addition, the pulmonary uptake of ^18^F-FDG on PET significantly correlated with global health scores and pulmonary physiologic measurements.

Initial data also suggested similar characteristics in IRD. Thus, Bellando-Randone et al. (81) demonstrated in patients with SSc that morphologically “positive” GGO segments showed an increased ^18^F-FDG uptake, suggesting the existence of an increased metabolic activity of GGO. According to the authors, these results might indicate that PET/CT may disclose an underlying inflammatory process, which cannot yet be evidenced by HRCT. In addition, Motegi presented similar results in patients with dermatomyositis (82). So far, there are no longitudinal data existing regarding the use of PET/CT in monitoring the course of ILD-IRD. Therefore, further research projects should be initiated to close this knowledge gap and examine the value of this technique in monitoring IRD-patients with known ILD. However, it has to be considered that PET/CT is mainly available in specialized centers and is characterized by high examination costs.

## Invasive diagnostic procedures

### Bronchoalveolar lavage

Bronchoalveolar lavage cell patterns and other characteristics can be an useful adjunct in patients with suspected ILD and a lack of confident UIP-pattern on HRCT (83). Although a normal BAL does not exclude ILD, the recognition of a predominantly inflammatory cellular pattern in the BAL can be helpful for the clinician to narrow the differential diagnosis of ILD, and perhaps lessens the need to proceed to more invasive procedures, such as surgical lung biopsy (25, 70–72). In addition, infection and malignancy can be excluded. Furthermore, BAL can be easily and safely performed and the risk of complications is lower than in transbronchial biopsy (84, 85). However, BAL should always be interpreted within the clinical context (medical history, physical examination, and imaging) (83, 86). In patients with suspected ILD, the recommended diagnostic examinations performed on BAL fluid include differential cell count, microbiological studies, and malignant cell cytology laboratory testing (see Table 6) (84, 85). The analysis of the cell patterns in BAL is also called immunological BAL. It has already been shown in the literature that the composition of immunological cells can vary depending on the CT pattern. Ryu et al. (87) reported an elevated lymphocyte count in NSIP compared to UIP in IPF-patients. In addition, differential diagnoses can be evaluated. Domagala described a high total cell count and increased pigmented macrophages in the differential diagnosis of RB-ILD (88). Furthermore, biomarkers measured in BAL could have a value in diagnosing ILD in IRD in the future [e.g., matrix metallopeptidase 9 (MMP-9) or CCL7] (89, 90). However, biomarkers are not yet established in the clinical routine. In Table 6 informative value of different BAL parameters in ILD were summarized.

**TABLE 6:** Informative value of different parameters in bronchoalveolar lavage (BAL) in ILD.

		Parameters	References	Informative value
Micro-biological		Bacteriological	No	• Exclude infection (86)
		Virological	No	
		Mycological	No	
		Parasitological	No	
Cytopathological			No	• Exclude malignancy (86)
Immunological	Differential cell count	Alveolar macrophages	>85% (86)	• Especially elevated in smoking-related ILD (86)
		Lymphocytes	10–15% (86)	• ↑↑ Detectable in various ILD (86) • Very high in hypersensitivity pneumonitis (86)
		Neutrophils	≤3% (86)	• ↑ Detectable in various ILD, but also typical in infection (86)
		Eosinophils	≤1% (86)	• ↑↑ Typical for eosinophilic disease (eosinophilic pneumonia, eosinophilic granulomatosis with polyangiitis) (86)
		Epithelial cells	≤5% (86)	• Proves the representativeness of the BAL (86)
		Blood cells	Ø	• ↑ Typical for diffuse alveolar hemorrhage (86)
	Lymphocytes subsets	CD4/CD8-ratio	<3.5 (86)	• ≥3.5 increase the likelihood of sarcoidosis (86)
		CD103^+^CD4^+^ T-lymphocytes	<40% (111)	• Elevated in ILD and decreased in sarcoidosis stage I (112–114)
		CD25^+^CD4^+^ T-lymphocytes	<10% (111)	• Elevated in smokers and COPD (115) • Relevant to prevent development of autoimmunity (116) • Increased percentage can occur reactively in the tissue of IRD-patients (116, 117)
		Natural killer cells	Unknown	• Higher percentage in hypersensitivity pneumonitis or organizing pneumonia (118–120) • Differences between the IRD can be expected (121)
		Natural killer T-cells	Unknown	• Higher percentage in hypersensitivity pneumonitis or organizing pneumonia (118–120) • Differences between various IRD can be expected (121)
		CD1a-positive cells	<5% (86)	• ≥5% increases the likelihood of Langerhans cell histiocytosis of the lung (86)
		B-cells	Unknown	• So far, no diagnostic relevance is known

It should be emphasized that there are no studies or evidence-based recommendations for immunological BAL in diagnosing IRD-ILD available. In addition, protocols for the standardized performance and analysis of a BAL are also lacking which would be essential to compare data. Considering these findings, the diagnostic, prognostic, and therapeutic value of BAL in IRD-ILD remains unclear (91). However, BAL can be a useful tool for the diagnostic evaluation of patients with suspected ILD. In addition, an immunological BAL might be helpful in differentiating between predominantly inflammatory or fibrotic CT patterns in IRD-ILD (23). Further research is necessary to verify these aspects.

In existing ILD in IRD the risk of pulmonary infections (bacteriological, virological, and mycological) is increased with an aggravation due to immunosuppressive therapy (92, 93). Moreover, infection can aggravate clinical symptoms as well as ILD and lead to a delay of the therapy (92, 94). Curtis et al. (94) reported an increased hospitalization rate for RA-ILD patients having a hospitalization for pneumonia in the last 12 months. In this context, BAL can be very helpful for the detection of pulmonary infections and planning an adequate treatment of ILD patients in clinical practice.

### Lung biopsy

**FIGURE 3:**
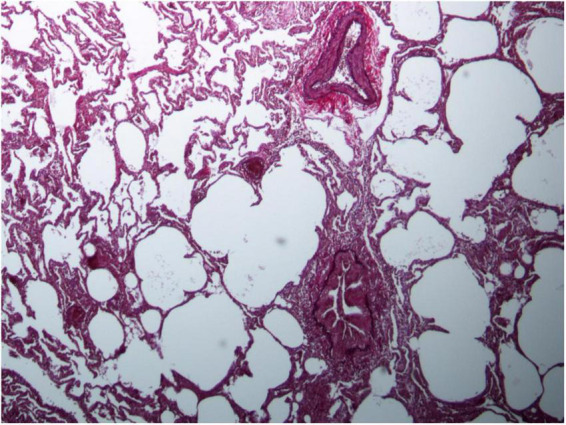
Lung biopsy in patient with MDA5-positive myositis representing with chronic inflammation and subpleural fibrosis.

Currently, biopsies are performed as open surgery or transbronchial biopsy (cryobiopsy or forceps biopsy) (see Figure 3). In IPF, surgical lung biopsy is still an important tool in a subset of patients who cannot be diagnosed based on clinical and imaging features alone (21, 67). According to the White Paper of the Fleischner Society, biopsy should be considered if the clinical context is indeterminate or the HRCT pattern is not definite or probable UIP (21, 67). As highlighted in the update of the ATS/ERS statement on the international classification of idiopathic interstitial pneumonias (IIP), a multidisciplinary approach does not lessen the importance of lung biopsy in the diagnosis of IIPs; rather, it defines the settings where biopsy is more informative than HRCT and those where biopsy is not needed (59).

There are no clear recommendations concerning the diagnostic value of lung biopsy in patients with IRD-ILD. Only a few publications mention the procedure as an additional option to confirm the diagnosis of ILD in IRD (53, 56). This may also be because patients are often considered to be too old or too sick for biopsy (68). Furthermore, major complications are more frequent in transbronchial biopsy (2.7%) than in BAL (0.12%) and in video-assisted thoracoscopic surgery (VATS) (up to 8%) even higher than in transbronchial biopsy (84, 85, 95). Major complications are especially pneumothorax, hemorrhage, respiratory depression, vasovagal episodes, and bronchospasm. In addition, histological knowledge usually does not change the planned therapy (68). In conclusion, lung biopsy can be helpful to exclude other reasons of ILD in IRD-patients and to achieve a higher diagnostic confidence.

## Biomarkers

### Autoantibodies

Inflammatory rheumatic disease are characterized by immunological laboratory parameters. Numerous autoantibodies are known and are often used to distinguish between the disease entities (e.g., anti-synthetase syndromes). Several serum autoantibodies associated with ILD are described in the literature. In RA, serum rheumatoid factor (RF) and anti-cyclic citrullinated peptide antibodies (anti-CCP) are associated with ILD (51, 96). Regarding patients with SSc, the presence of anti-topoisomerase I (Scl-70) antibodies is associated with progressive ILD and represent an independent risk factor (19). In myositis, myositis-specific and associated antibodies like anti-melanoma differentiation-associated protein 5 (anti-MDA-5) are an independent risk factor for rapid progressive ILD with high mortality, while anti-aminoacyl tRNA synthetase (anti-ARS) antibodies are associated with subacute ILD (97–99).

By using autoantibodies and other laboratory parameters, predictive models for mortality in IRD-patients with ILD were established. Gono et al. (25) reported a prognostic model in patients with myositis, based on serum levels of C-reactive protein (CRP), KL-6, and MDA-5 status. Similar correlations are known for other IRD.

### Other blood and non-blood biomarkers

In addition to the traditional autoantibodies, there are other blood and non-blood biomarkers which a potentially associated with IRD-ILD or have a prognostic factor. These biomarkers are the subject of research, but are not yet part of clinical routine.

In RA, different serum biomarkers (e.g., matrix metalloproteinases) and genetic polymorphisms (e.g., MUC5B) are reported with an association to ILD (100, 101). Furthermore, KL-6 is an experimental biomarker for the presence and progression of ILD in SSc (102, 103). There are also non-blood biomarkers, like the measurement of exhaled alveolar nitric oxide (NO), which correlates with the severity of ILD in SSc (104). In addition, there is evidence that intestinal dysbiosis can lead to increased systemic inflammation and increased extraintestinal involvement (105, 106).

## Combination of different diagnostic techniques

### Combination of clinical examination, pulmonary function test, and imaging

In clinical routine, the diagnosis of IRD-ILD is not made on the basis of a single examination, but is usually based on a combination of different examinations. As described in the literature, the combination of several PFT-parameters did not increase specificity without a significant loss of sensitivity in detecting ILD (49, 50).

Applying a simple clinical decision rule developed by Steele et al. (74) resulted in a sensitivity and specificity of 58.6–88.7% and 60.0%, respectively, in identifying ILD using physical examination or/and chest X-ray. The combination of chest X-ray or PFT (with FVC < 80% and FEV_1_/FVC > 70%) achieved a sensitivity and specificity of 60.5% and 77.3% with positive likelihood ratio (LR) 2.67 and negative LR of 0.51, respectively (74). Suliman et al. (49) and Bernstein et al. (51) showed sensitivities and specificities of 59.0–74.1% and 45.7–65.8%, respectively, with positive LR of 1.47–1.7 and negative LR of 0.36–0.6, by using a combination of FVC (<80%) and DLCO (<70 or <80%). According to the results gained by Hoffmann et al. (27), combining chest X-ray and PFT (DLCO < 80%) yielded a sensitivity and specificity of 95.2 and 38.7% with a negative LR of 0.12 in newly diagnosed IRD-patients. By combining (1) PFT (DLCO < 80%) and chest X-ray and (2) HRCT, Hoffmann et al. (27) provided a sensitivity and specificity of 95.2 and 77.4% with a negative LR of 0.06 in newly diagnosed IRD-patients.

### Combination of clinical examination, pulmonary function test, imaging, invasive techniques, and biomarkers

So far, there is no established algorithm for screening or monitoring of ILD in IRD available. Especially, studies do not exist yet, which examine the value of clinical examination, non-invasive diagnostics (PFT and imaging), invasive techniques (BAL or biopsy), and biomarkers.

Interpretation of these results would also be challenging, because it is difficult to define a gold standard. Perhaps a diagnosis developed within an ILD board on an interdisciplinary basis should become the gold standard. In the future, an algorithm for screening, monitoring, and prognostic assessment could be developed by combining different techniques (non-invasive, imaging, invasive, and biomarkers).

## Conclusion

Pulmonary complications in the form of ILD are among the most common and serious complications in IRD patients and may lead to significant morbidity and mortality. Given this background and available modern therapeutic options, early diagnosis of ILD in this patient population is essential.

For some examination techniques such as PFT, 6MWT, and HRCT that are firmly established in clinical practice and widely available, there is clear, broad evidence for their value in screening, monitoring, and prognostic assessment of ILD in IRD. MRI and PET/CT represent additional imaging modalities that may have a greater impact in the future by combining morphological and functional correlations. The results are promising, but further research is needed.

More evidence has also to be demanded on the use of invasive diagnostic methods such as BAL and lung biopsy. Both techniques can support the diagnosis of ILD in IRD, but their additional value [improvement of sensitivity, specificity, NPV, positive predictive value (PPV)] to HRCT remains unclear.

Furthermore, the possibility of a prognostic assessment is still very controversial. There is small evidence for biomarkers in predicting ILD and also on progression of known ILD. In addition, a clear definition of ILD from a rheumatological point of view is missing which would enable clinicians to interpret the available data. This is probably also difficult because there are sliding transitions from no changes to indeterminate ILA to ILA and finally to ILD. Consequently, it is very difficult to define gold standards for diagnosing ILD in IRD-patients.

For many other rheumatological diseases, the principle of “hit hard and early” already applies. Through the improved technology in HRCT, we will increase the proportion of patients with indeterminate ILA. At this point, it is important to differentiate between ILD and non-ILD patients at an early stage. This challenge cannot be addressed by HRCT alone. By combining PFT (in particular DLCO), cardiopulmonary stress tests and HRCT with invasive diagnostics and biomarkers, we might be able to develop an algorithm for screening, but also for monitoring and gaining prognostic information in the future. This would also allow a better management and the implementation of personalized, targeted therapies.

In the future, a combination of various methods (PFT, imaging, invasive diagnostics, and biomarkers) might allow the development of algorithms on the basis of which IRD-patients with ILD can be treated with a personalized, targeted medication.

## Author contributions

AP and TH designed the review, collected the data, wrote the manuscript, and revised the manuscript. GW, MF, UT, PO, CK, and NG edited and drafted the manuscript. All authors read and approved the final manuscript.
