# Supplementary material for: Diagnosing lung involvement in inflammatory rheumatic diseases—Where do we currently stand?
Source: Front Med (Lausanne). 2023 Jan 11;9:1101448. doi: 10.3389/fmed.2022.1101448 (PMC9874106; doi:10.3389/fmed.2022.1101448)
Supplement: Supplementary file 1 [file Data_Sheet_1.pdf]

Supplement: Diagnostic of interstitial lung disease in inflammatory rheumatic disease demonstrated on a patient with lung manifestation in systemic lupus erythematosus (SLE).

A 73-year-old woman was referred to hospital Jena based on arthralgia of the fingers, myalgia, weight loss and sicca symptomatic. The patient reported of mild shortness of breath (NYHA I) and mild cough. Physical examination did not show sklerosiphonia. The patient is long-time ex-smoker.

The laboratory tests revealed an elevated antinuclear antibody (ANA) titer (>1:1.280) as well as dsDNS antibodies. The basic laboratory parameter show no pathological changes (bloud count, inflammation parameter, urine examinations and liver or kidney parameter). Especially there were no consumption of the complement factors or antiphospholipid antibodies.

For further diagnostic evaluation an organ screening were performed (transthoracal echocardiography, lung function test including diffusing capacity of the lung for carbon monoxid (DLCO) and abdominal ultrasound). The lung function test revealed a DLCO < 80%, with otherwise normal values (see table 1), an additional chest X-ray revealed streaky condensations in the lower lobes (see figure 1A). The other examinations showed normal organ functions.

Table 1: Bodyplethysmography, spirometry and measurement of diffusing capacity of the lung at initial consultation.

**Parameter**	**% predicted (initial diagnosis)**	**% predicted (after induction therapy)**	**% predicted (long time follow up)**
Forced expiratory volume per second (FEV1)	103.7%	101.8%	106.8%
forced vital capacity (FVC)	98.8%	90.9%	102.3%
Total lung capacity (TLC)	88.6%	89.0%	88.9%
Diffusing capacity of the lung for carbon monoxide (DLCO)	63.0%	76.3%	82.6%
DLCO per ventilated volume (TLCO)	81.0%	88.8%	104.6%

High resolution computed tomography (HRCT) of the lungs showed distinct ground glass opacities (GGO) and reticulations in all lung segments, moreover decent bronchiectasis, but no significant honeycombing (see figure 1B). According to the Fleischner Society and the American Thoracic Society (ATS)/European Respiratory Society (ERS), the pattern was classified as NSIP (nonspecific interstitial pneumonia).

Figure 1: ***A*** Chest-X ray and ***B*** high resolution computered tomography (HRCT) of the lung at baseline as well as ***C*** HRCT after induction therapy with cyclophosphamide and glucocorticoids.

Under consideration the clinically and laboratory findings, the radiological findings were suspect for pulmonary involvement in systemic lupus erythematosus. There was no evidence for other organ involvement, so pulmonary involvement would determine the therapy.

To exclude differential diagnosis (e. g. microbiological, virological and malignancy) and evaluate inflammatory activity, a bronchoscopy with bronchoalveolar lavage (and additional option for biopsy) was performed. The bronchial system showed unremarkable, so that a biopsy was not initially performed. The bacteriological, virological and cytological examinations revealed negative results. In addition, an immunological analysis of the cell populations was performed by light microscopy and flow cytometry. The analysis showed an elevation of lymphocytes and neutrophils, but without evidence of infection. Furthermore, an increase of the CD4/CD8 quotient was observed (see table 2). Consequently, the results were interpreted as lymphocytic alveolitis.

Table 2: Analysis of bronchoalveolar lavage of the middle lobe.

**Parameter**		**Value**
Recovery		44ml
Total cell count		9.20 x 10^6 cells
Macrophages		57.0%
Lymphocytes		20.0%
	T-cells	82.7%
	B-cells	15.6%
	$CD4^+$ T-cells	66.2%
	$CD8^+$ T-cells	12.5%
	$CD4^+/CD8^+$	5.31
Neutrophils		15.0%
Eosinophils		3.0%
Bacteriology		negative
Virology		negative
Cytology		negative

In summary and considering the microbiological results, symptoms, imaging and laboratory diagnostics, the diagnosis of interstitial lung disease (ILD) in systemic lupus erythematosus (SLE) could be established after interdisciplinary discussion.

After the established diagnosis of ILD in SLE, an immunosuppressive induction therapy with cyclophosphamide and glucocorticoids was introduced, followed by maintenance therapy with azathioprine.

After the immunosuppressive induction therapy, the HRCT showed no significant change, with slightly reduced ground glass opacities (see figure 1C). The lung function test showed a normalization of the parameters (especially the DLCO) already after the initiated therapy, but also in the long-time follow-up of >4 years (see table 1).

The present case demonstrates the importance of multimodal diagnostic of ILD in IRD. Even patients with only minor changes in pulmonary function test may have significant ILD on HRCT at initial diagnosis which required an intensive immunosuppressive therapy. To exclude relevant differential diagnosis of HRCT patterns, further invasive diagnostic (e. g. bronchoscopy with bronchoalveolar lavage and biopsy) is necessary in suspected ILD, especially if lung involvement determined the therapy.
