# Supplementary figures and images for: Diagnosing lung involvement in inflammatory rheumatic diseases—Where do we currently stand?
Source: Front Med (Lausanne). 2023 Jan 11;9:1101448. doi: 10.3389/fmed.2022.1101448 (PMC9874106; doi:10.3389/fmed.2022.1101448)

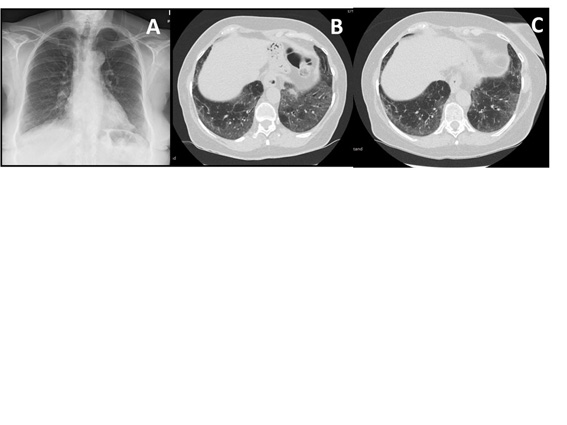

Supplement: Supplementary file 2 [file Image_1.JPEG]
